# In silico and expression analyses of fasciclin-like arabinogalactan proteins reveal functional conservation during embryo and seed development

**DOI:** 10.1007/s00497-019-00376-7

**Published:** 2019-09-09

**Authors:** Mário Costa, Ana Marta Pereira, Sara Cristina Pinto, Jessy Silva, Luís Gustavo Pereira, Sílvia Coimbra

**Affiliations:** 1 Departamento de Biologia, Faculdade de Ciências da Universidade do Porto, Porto, Portugal; 2 LAQV Requimte, Sustainable Chemistry, Universidade do Porto, Porto, Portugal; 3 GreenUPorto Sustainable Agrifood Production Research Centre, Porto, Portugal; 4 Dipartimento di Bioscienze, Università Degli Studi di Milano, Milan, Italy

**Keywords:** Fasciclin-like arabinogalactan proteins, Embryo development, Evolution, *Arabidopsis thaliana*, *Quercus suber*

## Abstract

**Key message:**

The fasciclin-like arabinogalactan proteins organization into four groups is conserved and may be related to specific roles in developmental processes across angiosperms.

**Abstract:**

Fasciclin-like arabinogalactan proteins (FLAs) are a subclass of arabinogalactan proteins (AGPs), which contain fasciclin-like domains in addition to typical AGP domains. FLAs are present across all embryophytes, and despite their low overall sequence similarity, conserved regions that define the fasciclin functional domain (FAS) have been identified, suggesting that the cell adhesion property is also conserved. FLAs in Arabidopsis have been organized into four subgroups according to the number and distribution of functional domains. Recent studies associated FLAs with cell wall-related processes where domain organization seemed to be related to functional roles. In Arabidopsis, FLAs containing a single FAS domain were found to be important for the integrity and elasticity of the plant cell wall matrix, and FLAs with two FAS domains and two AGP domains were found to be involved in maintaining proper cell expansion under salt stress conditions. The main purpose of the present work was to elucidate the expression pattern of selected FLA genes during embryo and seed development using RT-qPCR. AtFLA8 and AtFLA10, two Arabidopsis genes that stood out in previous microarray studies of embryo development, were further examined using promoter-driven gene reporter analyses. We also studied the expression of cork oak FLA genes and found that their expression partially parallels the expression patterns of the putative AtFLA orthologs. We propose that the functional organization of FLAs is conserved and may be related to fundamental aspects of embryogenesis and seed development across angiosperms. Phylogenetic studies were performed, and we show that the same basic four-subgroup organization described for Arabidopsis FLA gene classification is valid for most Arabidopsis FLA orthologs of several plant species, namely poplar, corn and cork oak.

**Electronic supplementary material:**

The online version of this article (10.1007/s00497-019-00376-7) contains supplementary material, which is available to authorized users.

## Introduction

Angiosperms are the vastest group of land plants (Wikström et al. 2001; Jiao et al. 2011). One of the most important characteristics of the flowering plants is the production of seeds. Seeds are consumed by humans, incorporated into animal feed and transformed into high-value-added products (FAO 2016). Seed development is the aftermath of pollination. Pollen lands on the stigma producing a pollen tube that transports the two sperm cells into the embryo sac for double fertilization: one fertilizes the egg cell to form the embryo, while the other fuses with the central cell to form the endosperm. Upon fertilization, the embryo and endosperm develop in such an interconnected way that embryogenesis is normally considered in the context of seed development. Seeds comprise three main components, embryo, endosperm and seed coat, and the coordinated development of each of these components is imperative for the correct formation of the mature seed.

The anatomy and genetics of embryo development have been extensively studied for the past few years, and several reviews are available (Capron et al. 2009; Boscá et al. 2011; Hove et al. 2015). The early stages of embryo development are essentially the same in practically all higher plants (Capron et al. 2009; Hove et al. 2015). In Arabidopsis, embryo development follows a predictable pattern, with the first division of the zygote being asymmetric. The precise development of the embryo is marked by the expression of specific regulatory genes, produced at nearly every stage of cell divisions (Hove et al. 2015). During seed development, as a consequence of the innumerous cell divisions taking place, there is a massive production of cell walls composed by a network of polymers where the major components are polysaccharides. The important role that the cell wall plays in cytokinesis, growth, and morphogenesis is well known and, therefore, genes that affect seed development through cell wall production are potential targets for crop improvement (Sechet et al. 2018).

*Quercus suber* is a core eudicot species, commonly known as cork oak. *Q. suber* is a monoecious evergreen tree with high economic importance in Europe (Duque-Lazo et al. 2018). It is found in the western region of the Mediterranean Basin (Lumaret et al. 2012), in both Europe (Italy, France, Spain, Portugal) and North Africa (Tunisia, Morocco, Algeria) (De Dato et al. 2018). The cork oak is part of the Natura 2000 network as an example of how sustainable human practices can favor natural habitats (Bugalho et al. 2011). This species has been implemented in reforestation strategies (Costa et al. 2017; Duque-Lazo et al. 2018), and it is a significant asset in agro-silvopastoral practices (Bugalho et al. 2012). The cork is a high-value-added resource for the industry (Capote et al. 2018; Testillano et al. 2018). Additionally, cork oak acorns are a supply of nutrient-rich food for wildlife, livestock and some human populations (Bugalho et al. 2012; Pausas et al. 2012). Due to the importance of seeds for mankind, one objective in modern seed biology is to map the molecular interactions regulating seed developmental processes (Martínez-Andújar et al. 2012).

Arabinogalactan proteins (AGPs) constitute a large and highly diverse class of heavily glycosylated proteins (Seifert and Roberts 2007; Pereira et al. 2014). They are typically rich in Pro/Hyp, Ala, Ser and Thr, *O*-glycosylation motifs, and most members have a glycosylphosphatidylinositol (GPI) anchor addition sequence. AGPs have been reported to function in sexual reproduction in both Arabidopsis (for relevant reviews see Pereira et al. 2015 and Su and Higashiyama 2018) and cork oak (Costa et al. 2015; Lopes et al. 2016). Moreover, AGPs temporal and spatial expression patterns during embryo development were shown, indicating their potential relevance for seed development in both Arabidopsis (Hu et al. 2006) and *Nicotiana tabacum* (Qin and Zhao 2007).

The fasciclin-like arabinogalactan proteins (FLAs) are a subclass of AGPs implicated in plant growth and development. They have been identified in a vast range of plants such as Arabidopsis, cabbage, rice, wheat, cotton, poplar, loblolly pine and zinnia (Dahiya et al. 2006; Faik et al. 2006; Johnson et al. 2003; Lafarguette et al. 2004; Loopstra et al. 2000; Yang et al. 2005; Huang et al. 2008). FLAs are characterized by containing one or two FAS1 (fasciclin-like) domains and at least one AGP module. FAS1 domains are extracellular polypeptide modules that, at least in animal and algal cells, have experimentally confirmed roles in cell adhesion (Bastiani et al. 1987; Huber and Sumper 1994; Park et al. 2004, 2009; Thapa et al. 2005; Jung et al. 2007). These domains were originally identified in the structure of insect fasciclin I protein (Bastiani et al. 1987) homologs of which are believed to be present in most organisms, including bacteria (Ulstrup et al. 1995). Despite the low amino acid sequence similarity among FAS1 domains, two conserved short amino acid segments (H1 and H2 of about 10 amino acids each) can be identified in multiple alignments of FAS1 sequences (Kawamoto et al. 1998; Johnson et al. 2003).

The sequence similarity, the number and position of FAS1 and AGP domains, and the presence of GPI anchor addition sequence were used to classify the Arabidopsis FLAs in four groups (Johnson et al. 2003). Briefly, group A includes FLAs with one FAS1 domain flanked by AGP modules and a C-terminal GPI anchor signal. Group B clusters FLAs with one AGP module flanked by FAS1 domains and no GPI anchor signal; members in group C have either one or two FAS1/AGP module arrangement, and they all have the C-terminal GPI anchor signal. Other FLAs with no obvious relationships with groups A–C are placed in group D (Johnson et al. 2003). This chimeric nature of FLAs seems to be unique to plants and together with the fact that plants contain the greatest number of FAS1-containing protein genes, when compared to other organisms (Seifert 2018), makes this gene family even more intriguing.

By analogy to their animal counterparts, FLAs are putative cell adhesion molecules, but a clear picture for the role of these proteins in plants is lacking. AtFLA4 (also known as SALT OVERLY SENSITIVE 5/SOS5) was identified during a screen for mutants related to salt stress response (Shi et al. 2003). Although conditional root swelling was the most dramatic phenotype of *atfla4*, the mutant also showed thickened hypocotyls and inflorescence stems, larger leaves and shorter siliques, indicating that AtFLA4 can act non-conditionally throughout the plant. Other defects in *atfla4*, both in the attachment of the mucilage that protrudes from the seed coat upon hydration to the seed surface and in the roots, which lacked the pectin-rich middle lamella, are, however, clearly related to cell adhesion (Harpaz-Saad et al. 2011; Griffiths et al. 2014; Showalter and Basu 2016). At the molecular level, FLA4/SOS5 is reported to be a ligand of two cell wall leucine-rich repeat receptor-like kinases (FEI1/FEI2; Xu et al. 2008).

FLAs have also been shown to be important for their role in secondary cell wall formation, especially in Arabidopsis, cotton and poplar (Ito et al. 2005; Liu et al. 2008; Huang et al. 2008, 2013; Kaku et al. 2009; Hobson et al. 2010). Secondary cell walls are undoubtedly the most abundant fiber and energy feedstock on Earth, and its major constituents are among the most abundant biopolymers. Arabidopsis FLA11 and FLA12 were shown to be associated with secondary cell wall biosynthesis, and a *fla11 fla12* T-DNA double null mutant revealed reduced stem tensile strength (Ito et al. 2005; MacMillan et al. 2010). Parallel functions were also described for their respective orthologs in other plant species such as *Populus trichocarpa* (Wang et al. 2017). Recent studies (Li et al. 2016) indicated the existence of molecular interactions between cotton FLAs and secondary cellulose synthase GhCesA8, supporting the proposition that group A FLAs may physically interact with the cellulose synthase complex (Seifert 2018). Another Arabidopsis FLA gene, *AtFLA3*, was demonstrated to be pollen-specific, and *atfla*3 loss-of-function mutants showed partial pollen abortion during the transition from uninucleate microspores to bicellular pollen as well as possible defects in intine formation (Li et al. 2010).

As part of our contribution to the study of Arabidopsis and cork oak sexual reproduction (Costa et al. 2015; Lopes et al. 2016), we describe in the present work the developmental expression pattern of different FLA genes in four different species: Arabidopsis, poplar, corn and cork oak, concluding that the expression patterns of this class of AGPs is conserved across these species and is potentially important for embryogenesis and seed development. The first part of the work presented focuses on the in silico analysis of FLAs gene expression data and phylogeny. The second part is dedicated to validate these analyses for a group of specific FLAs important for seed development in Arabidopsis and cork oak.

## Materials and methods

### Plant material and growth conditions

Seeds of wild-type (wt) *Arabidopsis thaliana* (L.) Heynh., ecotype Columbia (Col-0) were obtained from the Nottingham Arabidopsis Stock Centre (NASC, UK). Seeds were stratified for 2 days at 4 °C in the dark and sown directly onto soil. Plants were grown in a growth cabinet, under continuous light at 18–21 °C, with 50–60% relative humidity. Developing seeds were collected in groups for RNA extraction and histological studies, according to their developmental stages (Goldberg et al. 1994). Three stages were considered: globular and heart-shaped (S2), torpedo (S3), walking stick and mature (S4) embryo. The developing seeds were isolated from the siliques under a stereo microscope, using hypodermic needles. An additional stage, S1, corresponding to flowers with unfertilized ovules (stage 12 of Smyth et al. 1990), was prepared for RNA extraction.

Flowers and young acorns of cork oak trees (*Quercus suber* L.) in different stages of development were collected at several time points between May and September, from two natural populations in the Porto area. Flowers and maturing acorns were selected according to the developmental stages described in Miguel et al. (2015). Maturing acorns were dissected under a magnifying glass, and the embryos were isolated and grouped according to their maturation stage. Three stages were established: globular and heart-shaped (S2), cotyledon (S3) and mature (S4). An additional stage was established with receptive ovules (S1). In samples of stages S1 and S2, the integuments were included. S4 samples were prepared by sectioning the seeds approximately 5 mm around the embryo axis to reduce the cotyledon influence on the total RNA extract.

### RNA extraction and cDNA synthesis

Total RNA from Arabidopsis flowers and seeds was extracted using PureZol™ RNA Isolation Reagent (Bio-Rad), following the instructions manual. About 300 Arabidopsis and 15 Quercus suber embryos were used per sample. Total RNA from cork oak embryos was isolated using the method described in Gambino et al. (2008) with some minor adaptations. Tissue samples (100 mg) were ground with a plastic pestle in a 1.5-mL microtube containing 900 μL of extraction buffer (2% cetyltrimethylammonium bromide [CTAB], 2.5% polyvinyl pyrrolidone Mw 40.000 [PVP-40], 2 M NaCl, 100 mM Tris–HCl pH 8.0, 25 mM EDTA and 2% β-mercaptoethanol) and incubated at 65 °C for 10 min. The homogenate was subjected to centrifugation at 12,000 × *g* for 5 min. The supernatant was mixed with an equal volume of chloroform:isoamyl alcohol (24:1 v/v) and centrifuged at 12,000 × *g* for 10 min at 4 °C. Nucleic acids were precipitated from the supernatant in 3 M LiCl during 30 min on ice and centrifuged at 18,000 × *g* for 20 min at 4 °C. The resulting pellet was resuspended in 30 μL SSTE buffer (10 mM Tris–HCl pH 8.0, 1 mM EDTA, 1% SDS, 1 M NaCl), mixed with 300 μL of chloroform:isoamyl alcohol mixture and centrifuged at 11,000 × *g* for 10 min at 4 °C. The RNA in the aqueous phase was precipitated by adding 600 μL of ice-cold isopropanol. Finally, the RNA was pelleted by centrifugation at 18,000 × *g* for 15 min at 4 °C. Sedimented RNA was washed with 70% ethanol, air-dried and dissolved in nuclease-free PCR-grade water. After quantification, 1 ng of total RNA was included in cDNA synthesis reaction mixtures. For both cork oak and Arabidopsis cDNA synthesis, RevertAid First Strand cDNA Synthesis Kit (Thermo Scientific) and oligo (dT)_18_ primers were used for the reverse transcription reaction. All procedures were performed according to Pereira et al. (2016).

### Arabidopsis FLA gene expression heat map of developing seeds

The expression levels of Arabidopsis FLA genes were scrutinized from the embryo development 21 k Affymetrix GeneChip assay dataset, available at the Seed gene network website (Belmonte et al. 2013; probe data in Supplemental Table 1), from the EFPbrowser server (Winter et al. 2007) and from the “AtGenExpress: Expression Atlas of Arabidopsis Development” (Schmid et al. 2005). Heat maps for the seed, embryo and seed coat were obtained using the Morpheus software. Arabidopsis FLA gene expression pattern similarity was estimated using the Euclidean Average distance method with a 0.5 cutoff (https://software.broadinstitute.org/morpheus).

### In silico FLA gene identification

Glycoprotein identification and classification software Protclass (Lichtenberg et al. 2012) was used to identify putative FLAs in four angiosperms with curated transcriptomes (*Arabidopsis thaliana*—GCA_000001735; *Zea mays*—GCA_000005005; *Populus trichocarpa*—GCA_000002775; *Quercus suber*—GCA_002906115). To improve Protclass prediction, the functional domain classification of putative FLA homologs was evaluated using SMART (Letunic and Bork 2017), and accordingly, protein sequences without a FLA typical domain were discarded. The remaining putative FLA sequences were aligned with MEGA X (Kumar et al. 2018), and to improve the alignment quality, only the mature protein core was considered. The presence of signal peptide and GPI anchor addition sequences were predicted with SignalP 4.1 Server (Nielsen 2017) and big-PI plant predictor (Eisenhaber et al. 2003), respectively. The presence of conserved functional domains was also used has a criterion to discriminate between potential homologs.

### Phylogenetic trees

Phylogenetic trees for the analysis of FLA genes were obtained with MEGA X software (Kumar et al. 2018), using the UPGMA method. The bootstrap consensus tree inferred from 500 replicates was taken to represent the evolutionary history of the identified putative FLAs. The percentage of replicate trees in which the associated taxa clustered together in the bootstrap test (500 replicates) are shown next to the branches. The evolutionary distances were computed using the Poisson correction method and are in the units of the number of amino acid substitutions per site. The rate variation among sites was modeled with a gamma distribution (shape parameter = 1). This analysis involved 126 amino acid sequences. All ambiguous positions were removed for each sequence pair (pairwise deletion option). There were a total of 766 positions in the final dataset. The Arabidopsis, and the Arabidopsis vs cork oak, FLA trees were obtained by the neighbor-joining method. This analysis involved 21 amino acid sequences. All ambiguous positions were removed for each sequence pair (pairwise deletion option). There were a total of 273 positions in the final dataset.

### Quantitative real-time PCR (qRT-PCR)

To select specific gene regions for qRT-PCR primer design, the coding sequences associated with accessions of cork oak and Arabidopsis were blasted (using blastp algorithm) against non-redundant protein sequence database restricted to the respective organism (NCBI taxid:58331 and taxid:3702). Output sequences were aligned against the target accession coding sequence using ClustalW (Larkin et al. 2007), and regions with no significant sequence identity were identified to generate amplicons with approximately 250 bp with the aid of Primer3Plus (Untergasser et al. 2012). Oligonucleotide primers (Supplemental Tables S2, S3) and amplicons were blasted against the NCBI taxid: 58331 database (using blastn algorithm) to confirm their specificity. PP2AA3 (XM_024062388), ACT7 (XM_024037498.1) (Sobral and Costa 2017), ACT2 (AT3G18780) (Takeuchi and Higashiyama 2012) and ACT8 (AT1G49240) were used as reference genes. Serial tenfold dilutions of cDNA from cork oak and Arabidopsis were used to determine primer efficiency curves. cDNA was amplified using the SSoFast^®^ SYBR Green Supermix in a CFX96 qRT-PCR Detection System (Bio-Rad). Three independent biological replicates were analyzed by quantitative RT-PCR for both Arabidopsis and cork oak samples. After the first denaturation step at 95 °C for 3 min, samples were run for 45 cycles of 10 s at 95 °C and 30 s at 58 °C. After each run, dissociation curves were obtained to check for amplification specificity by heating the samples from 60 to 95 °C in 0.5 °C increments. At the end of the PCR cycles, data were analyzed with Bio-Rad Software CFX Maestro™ 1.1, using the Livak calculation method (Livak and Schmittgen 2001).

### DNA construct generation and plant transformation

Genomic sequences corresponding to the upstream promoter regions of two FLA genes, *AtFLA8* and *AtFLA10* were amplified using Phusion DNA polymerase (Thermo Scientific), with the primer pairs described in Supplementary Table S4. The amplified upstream promoter regions spanned from the end of the untranslated region of the upstream neighboring gene down to the start codon of *AtFLA8* or *AtFLA10*. The size of the amplified region was kept around 3-3.3 kbp. PCR amplification products of the *AtFLA8* and *AtFLA10* promoter regions were first cloned into pENTR™/D-TOPO (Invitrogen) and then transferred into a Gateway-compatible version (Zheng et al. 2011) of the pGreenII-based vector NLS:3GFP:NOSt (Takada and Jürgens 2007), named pGII_GW:NLS:3GFP:NOSt. All constructs were confirmed by DNA sequencing. The pGreenII-based expression vectors were introduced into *Agrobacterium tumefaciens* GV3101 harboring the pGreenII helper plasmid pSOUP. Expression vectors were used to transform Arabidopsis (Col-0) by the floral dip transformation method (Clough and Bent 1998).

### Preparation of plant material for microscopy

Arabidopsis ovules and developing seeds were cleared as described by Brambilla et al. (2007) with minor changes. Flowers and siliques were fixed in 10% acetic acid in ethanol at 4 °C for 16 h, then washed in 90% and 70% (v/v) ethanol, each for 10 min, and finally cleared in a solution containing 160 g of chloral hydrate, 100 mL of water and 50 mL of glycerol. Using hypodermic needles (0.4 × 20 mm), the plant material was dissected under a stereo microscope Nikon SMZ1000 and observed using an upright Zeiss Axiophot D1 microscope equipped with differential interference contrast optics. Images were captured with an Axiocam MRc3 camera (Zeiss) using the Zen lite software (Zeiss). pFLA8:GFP and pFLA10:GFP ovules and developing seeds were dissected as described and observed using a Nikon Eclipse Ti-S microscope. Images were captured on a DS-Ri2 camera (Nikon) using the NIS-Elements Basic Research microscope imaging software (Nikon). GUS assays were performed on inflorescences as described by Liljegren et al. (2000). Following GUS staining, tissue samples were cleared and inspected as above.

Samples of immature acorns and receptive ovules from all four developmental groups of cork oak were fixed and imbedded in London resin, as described in Coimbra et al. (2007). Two-micrometer-thick sections were obtained with a Leica UC7 ultramicrotome equipped with Ralph glass knives and placed on glass slides. Sections were stained with toluidine blue dye (1% toluidine blue O in 1% sodium borate) for 5 min at 45 °C, followed by 10 s in a solution of 0.1% Safranin O. Slides were then left to dry and mounted with DPX mounting medium. Image acquisition was performed with Cellsens software through an Olympus DP73 digital camera mounted on a Leica DMlb upright microscope equipped with N-Plan Lens.

## Results

### Phylogenetic relationships among fasciclin-like arabinogalactan proteins

**Fig. 1:**
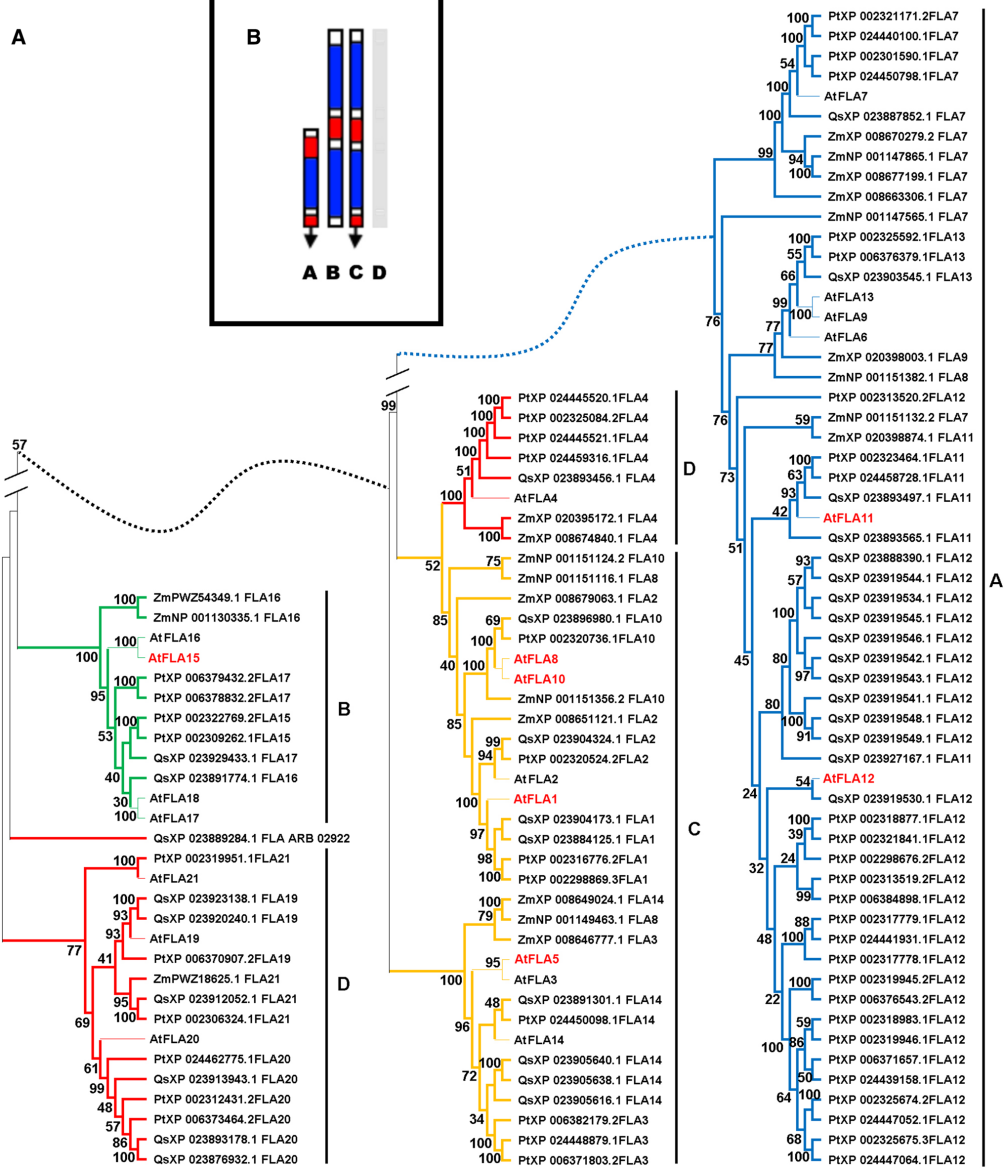
Bootstrap consensus tree of Arabidopsis (At), cork oak (Qs), poplar (Pt) and maize (Zm) putative fasciclin-like arabinogalactan (FLAs). The phylogenetic relationships of the putative FLAs were inferred using the UPGMA method. The UPGMA phylogenetic tree shows four distinct clusters (A (blue line), B (green line), C (yellow line) and D (red line)) (**A**) that correlates with the FLA classification model of Johnson et al. (2003) based in the arrangement and number of functional domains; AGP domains are shown in red, Fasciclin domains in blue, GPI anchor black arrows (**B**). The phylogenetic analyses were conducted with MEGA X

The phylogenetic relationships between putative FLAs of four divergent angiosperms (Fig. 1A), Arabidopsis (At; Brassicaceae), maize (Zm; Poaceae), cork oak (Qs; Fagaceae) and poplar (Pt; Salicaceae), were analyzed. All these species have relatively well-annotated genomes, and their transcriptomes have been previously characterized. The putative FLA sequences from these distantly related species cluster into four groups following the classification proposed for Arabidopsis FLAs (Johnson et al. 2003; Fig. 1B). Globally, putative orthologs of Arabidopsis FLAs could be found in the other three species. In particular, abundant variants of AtFLA12 were found in cork oak and poplar genomes. Conversely, the maize genome does not seem to have a clear putative AtFLA12 ortholog, but contains many sequences with high amino acid sequence identity to AtFLA7.

The FLA group B (Fig. 1A, in green) sets apart from all other groups. Members of this clade are low in number and form a tight cluster. However, the maize members of this group standout by branching off the main cluster.

Group C FLAs (Fig. 1A, in yellow) form two distinct subclades separating the FLAs with one FAS-AGP module (AtFLA3, PtFLA3, ZmFLA3, AtFLA5, AtFLA14, QsFLA14, PtFLA14, ZmFLA14) from the FLAs with two FAS-AGP modules (AtFLA1, PtFLA1, QsFLA1, AtFLA2, PtFLA2, QsFLA2, ZmFLA2, AtFLA8, ZmFLA8, AtFLA10, PtFLA10, QsFLA10, ZmFLA10). The subclades formed within group C contain the putative orthologs of the Arabidopsis members. A few maize FLAs do not follow this trend and are more closely related to their putative paralog, than with their orthologs [ZmFLA10 (NP 001151124.2), ZmFLA8 (NP 001151116.1), ZmFLA2 (XP 008679063.1), ZmFLA14 (XP 008649024.1), ZmFLA8 (001149463.1), ZmFLA3 (XP 008646777.1)].

Within group D (Fig. 1A, in red), it is possible to observe the formation of two subclades composed by the respective AtFLA20 and AtFLA4 putative orthologs. AtFLA21 and PtFLA21 (XP 002319951.1) diverge from the other group D FLAs, particularly the remaining FLA21 sequences. QsFLA21, ZmFLA21 and PtFLA21 (XP 002306324.1) cluster together in a subclade with AtFLA19. Within this group, the maize genome contains closely related genes only for AtFLA21 (Zm PWZ18625.1) and for AtFLA4 (Zm XP020395172.1 and Zm XP008674840.1).

### Microarray data of Arabidopsis FLAs during embryogenesis

**Fig. 2:**
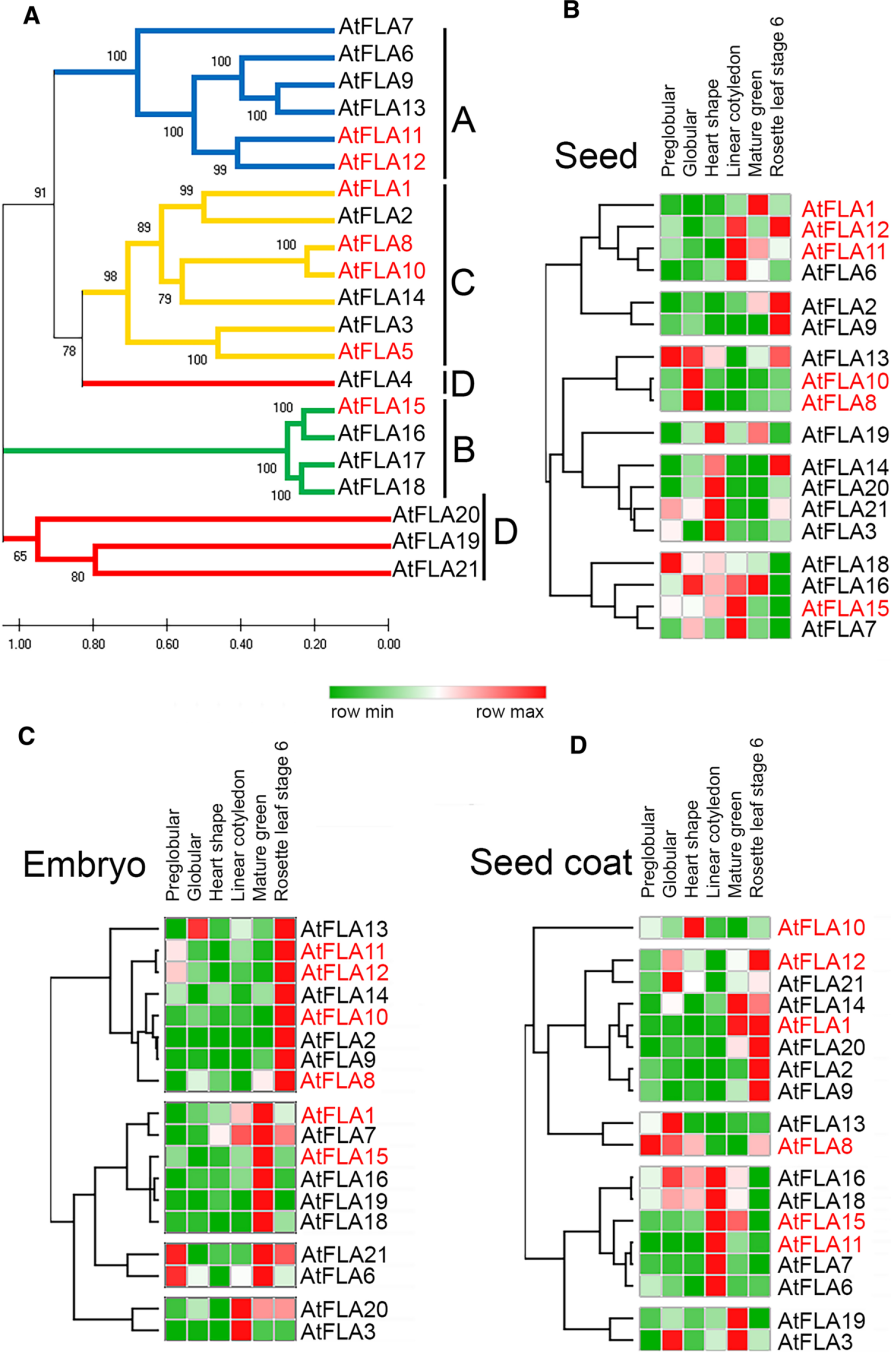
Neighbor-joining phylogenetic tree of Arabidopsis FLAs (**A**) and their microarray-based expression during seed development (**B**–**D**). The family of Arabidopsis FLAs is comprised of 21 members that can be structurally organized into four groups: A (blue line), B (green line), C (yellow line) and D (red line) (**A**). The phylogenetic relationships of the 21 FLA protein sequences were inferred using the neighbor-joining method. The heat maps compiling the normalized expression values of FLAs in the developing seed (**B**), embryo (**C**) and seed coat (**D**) are shown. Gene expression values are ranked from maximum (red) to minimum (green), and pattern similarity was estimated using the Euclidean average distance (https://software.broadinstitute.org/morpheus)

The Arabidopsis FLA family is composed of 21 members (Fig. 2A); for the ones with microarray data available in public databases, this was further analyzed. According to available microarray data, members of the same FLA group often show very different expression patterns (Fig. 2). The clustering of FLA genes based on the expression level in the seed (Fig. 2B), embryo (Fig. 2C) and seed coat (Fig. 2D) do not reflect the classification proposed by Johnson et al. (2003) (Fig. 2A). Therefore, it is not possible to predict an expression profile of a particular FLA group during seed development. Only group B AtFLA15, AtFLA16 and AtFLA18 seem to have a similar expression pattern in the seed, embryo and seed coat (Fig. 2B–D). A few FLA genes reach their maximum expression during embryo development (Fig. 2C), but only in specific stages of embryo maturation.

AtFLA15 is expressed during all seed developmental stages (Fig. 2B–D). In the embryo, it reaches the highest expression levels during the mature green embryo stage (Fig. 2C). In the seed coat, the maximum expression occurs in the linear cotyledon stage (Fig. 2D).

AtFLA8 and AtFLA10 are putative paralogs (Fig. 2A). AtFLA8 is expressed mostly in the globular stage and its expression decreases as the seed matures (Fig. 2C). According to microarray data, AtFLA8 expression in the seed coat (Fig. 2D) contributes the most to the overall seed expression level (Fig. 2B). AtFLA10 expression is more restricted, and its maximum expression occurs in the seed coat and during the heart-shaped stage (Fig. 2D). Another pair of closely related paralogs is AtFLA11 and AtFLA12, which have similar expression profiles during seed development (Fig. 2B), and both showed a somewhat relevant expression, specifically at the preglobular stage of embryo development (Fig. 2C), but have clearly different expression patterns during the seed coat development (Fig. 2D).

### FLA8 and FLA10 expression during seed development

**Fig. 3:**
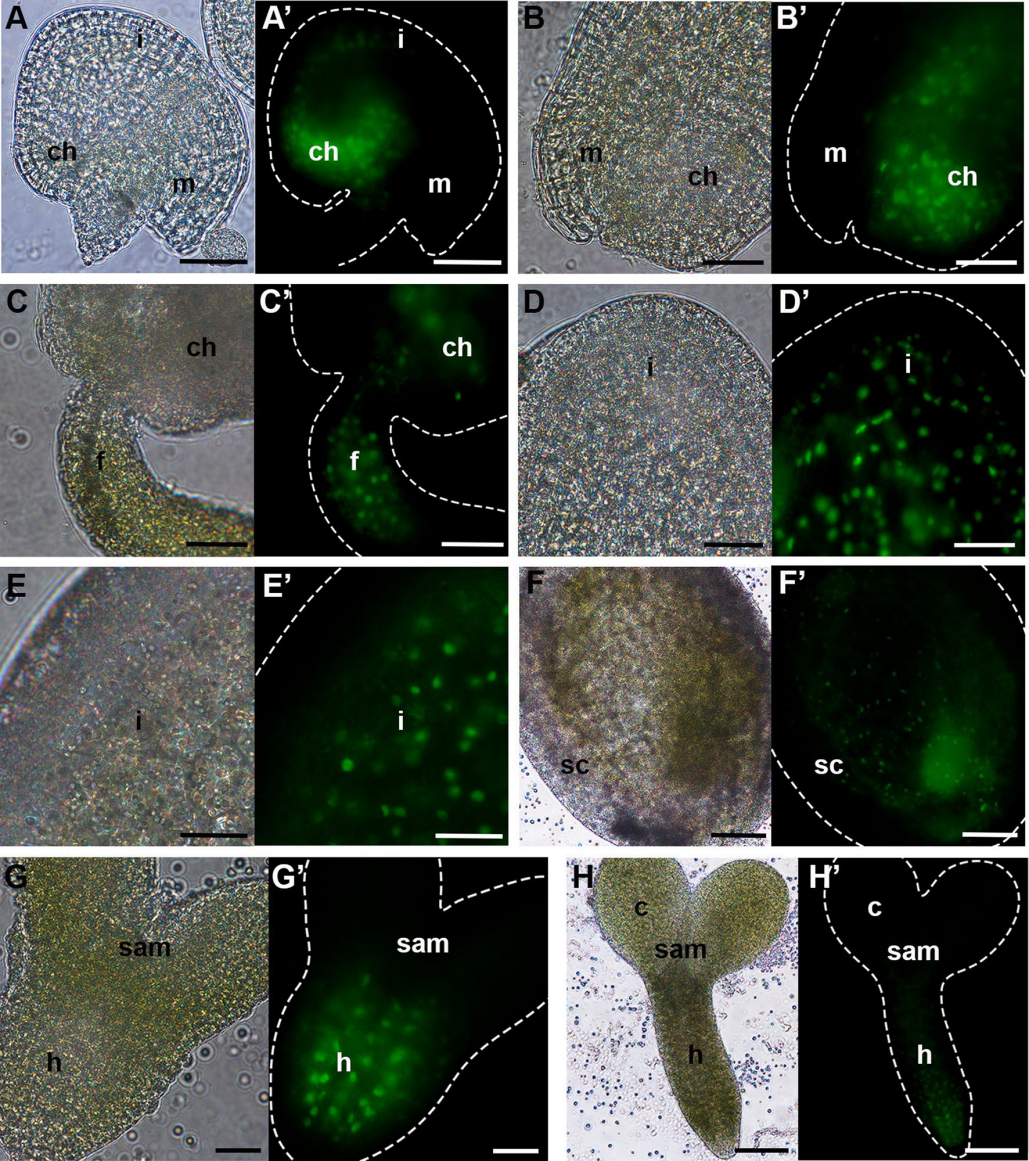
pFLA8-GFP expression pattern during seed development in Arabidopsis. **A**, **H** Bright-field microscopy images; **A′**, **H′** fluorescence microscopy images. **A**, **A′** unfertilized ovule; **B**, **C′** pre- and globular stage; **D**, **D′** heart stage; **E**, **E′** linear cotyledon stage; **F**, **F′** walking stick stage; **G**, **G′** heart-shaped stage embryo; **H**, **H′** walking-stick-stage embryo. c, cotyledon; ch, chalaza; f, funiculus; h, hypocotyl; i, integuments; m, micropyle; SAM, shoot apical meristem; sc, seed coat. Scale bars: 40 µm (**A**–**E**, **G**); 100 µm (**F**, **H**)

Previous studies based on microarray data (unpublished) suggested AtFLA8 and AtFLA10 to play important roles during seed development. These two were chosen to validate the transcriptomic data. AtFLA8 and AtFLA10 promotor regions fused with the GFP reporter gene were used to determine their spatiotemporal expression pattern during seed development. GFP expression driven by AtFLA8 promoter in the unfertilized ovule localized at the chalaza, in the chalazal region of the integuments (Fig. 3A′) and in the funiculus (Supplementary Figure 1A). During preglobular and globular stages of the developing seed, the fluorescence signal was found in the seed coat integuments and remained in the chalaza (Fig. 3B′, C′). Between the heart-shaped and walking stick stages of seed development, GFP was found to be expressed in the seed coat outer integuments (Fig. 3D′–F′). In embryos at the heart-shaped stage, GFP expression was detected in the hypocotyl cells surrounding the vascular bundle (Fig. 3G′). The fluorescence signal continued to be present in the embryo at the linear cotyledon and walking stick stages (Fig. 3H′).

**Fig. 4:**
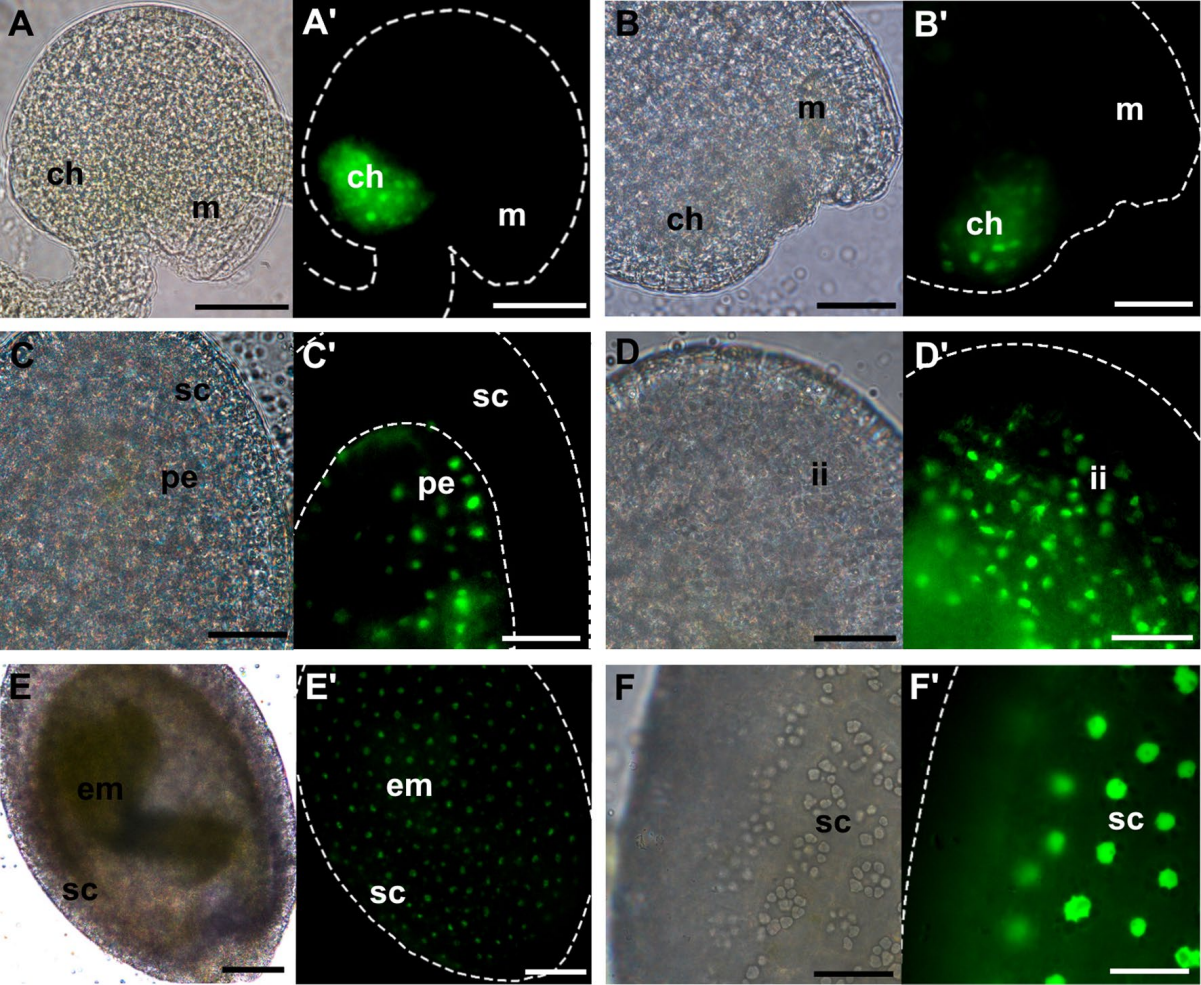
pFLA10-GFP expression pattern during seed development in Arabidopsis. **A**–**F** Bright-field microscopy images; **A′**–**F′** fluorescence microscopy images. **A**, **A′** unfertilized ovule; **B**, **C′** pre- and globular stage; **D**, **D′** heart-shaped stage; **E**, **E′** bent-cotyledon stage; **F**, **F′** mature seed. ch, chalaza; em, embryo; ii, inner integument; pe, peripheral endosperm; m, micropyle; sc, seed coat. Scale bars: 40 µm (**A**–**D**, **F**); 100 µm (**E**)

pFLA10-GFP was found to be expressed in the chalaza of unfertilized ovules (Fig. 4A′). Between the preglobular and globular stages, GFP signal was observed in the chalaza (Fig. 4B′) and peripheral endosperm (Fig. 4C′). From the heart-shaped to linear cotyledon stages, AtFLA10 promoter drove the expression of GFP in the seed coat integuments (Fig. 4D′). During later stages of seed development, the signal was observed in the outer layer of the seed coat (Fig. 4E′, F′). In the embryo, during the different stages of development, GFP signal was never detected (Supplementary Figure 1B).

### Expression pattern of putative Arabidopsis and cork oak FLA orthologs

**Fig. 5:**
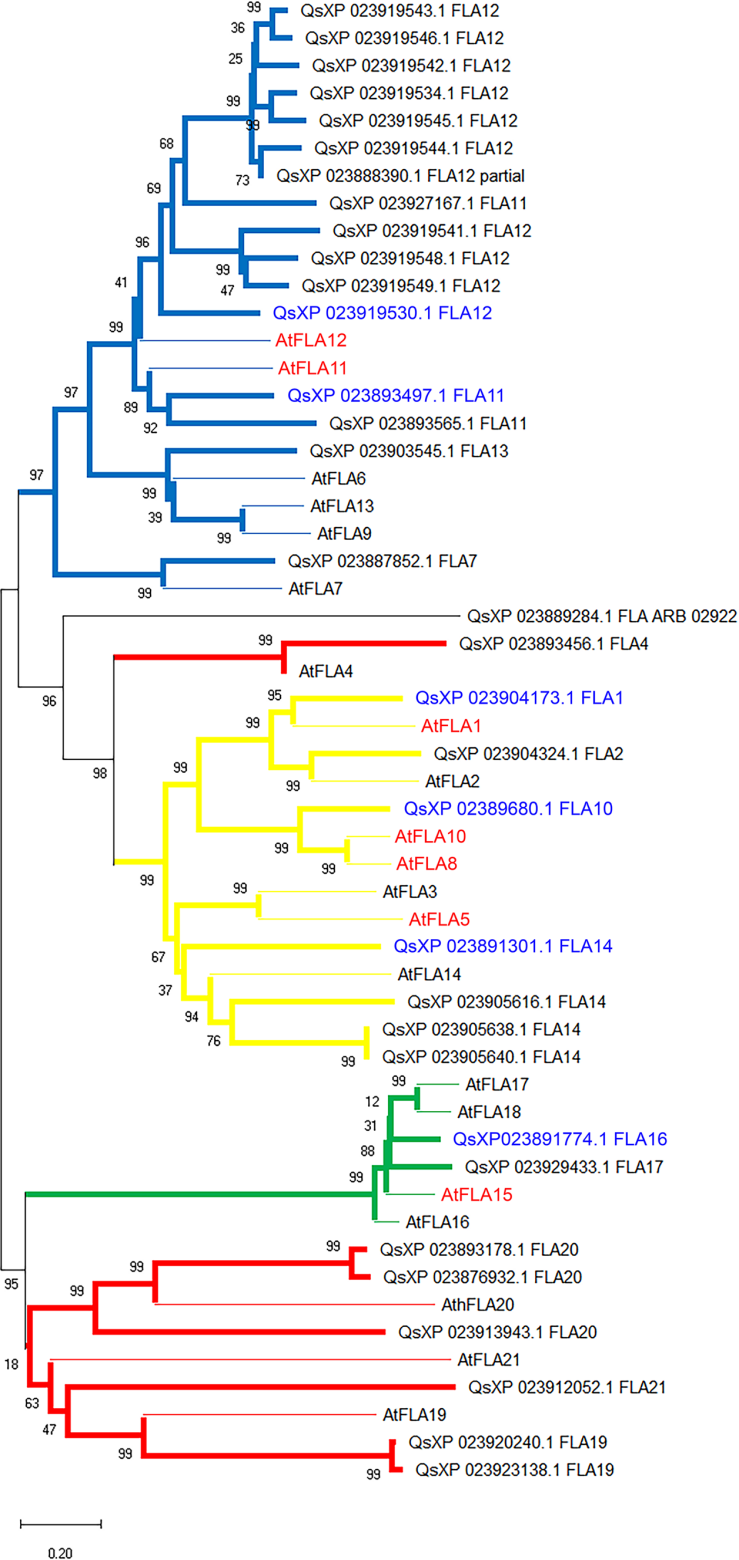
Phylogenetic relationships between Arabidopsis and cork oak FLAs. The 21 Arabidopsis FLAs were aligned with all known putative FLAs from the cork oak transcriptome (GCA_002906115), and the phylogenetic relationships were inferred using the neighbor-joining method. Evolutionary analyses were conducted in MEGA X

Putative orthologs for most Arabidopsis FLAs can be found in the cork oak genome (Fig. 5). The main difference between Arabidopsis and cork oak seems to be the relative number of members in each FLA class. Cork oak genome is enriched in group A FLAs, most of them related to AtFLA12, whereas group C FLAs are proportionally more abundant in Arabidopsis. AtFLA1 is more related to its ortholog in cork oak (Qs XP_023904173.1 FLA1) than with its closest paralog AtFLA2. The same occurs with AtFLA11 (QS XP_023893497 FLA11) and AtFLA12 (QS XP_023919530 FLA12). Nevertheless, the selection of a proper ortholog for AtFLA8 and 10 is difficult since the putative ortholog QsFLA10 (Qs XP_02896980) is equally distant from the two. No clear ortholog seems to be identifiable for AtFLA5 and AtFLA3 (the closest relative for both in the cork oak genome is QsFLA14—XP_023905616), and likewise, AtFLA15 probable ortholog is QsFLA16 (XP_023891774).

**Fig. 6:**
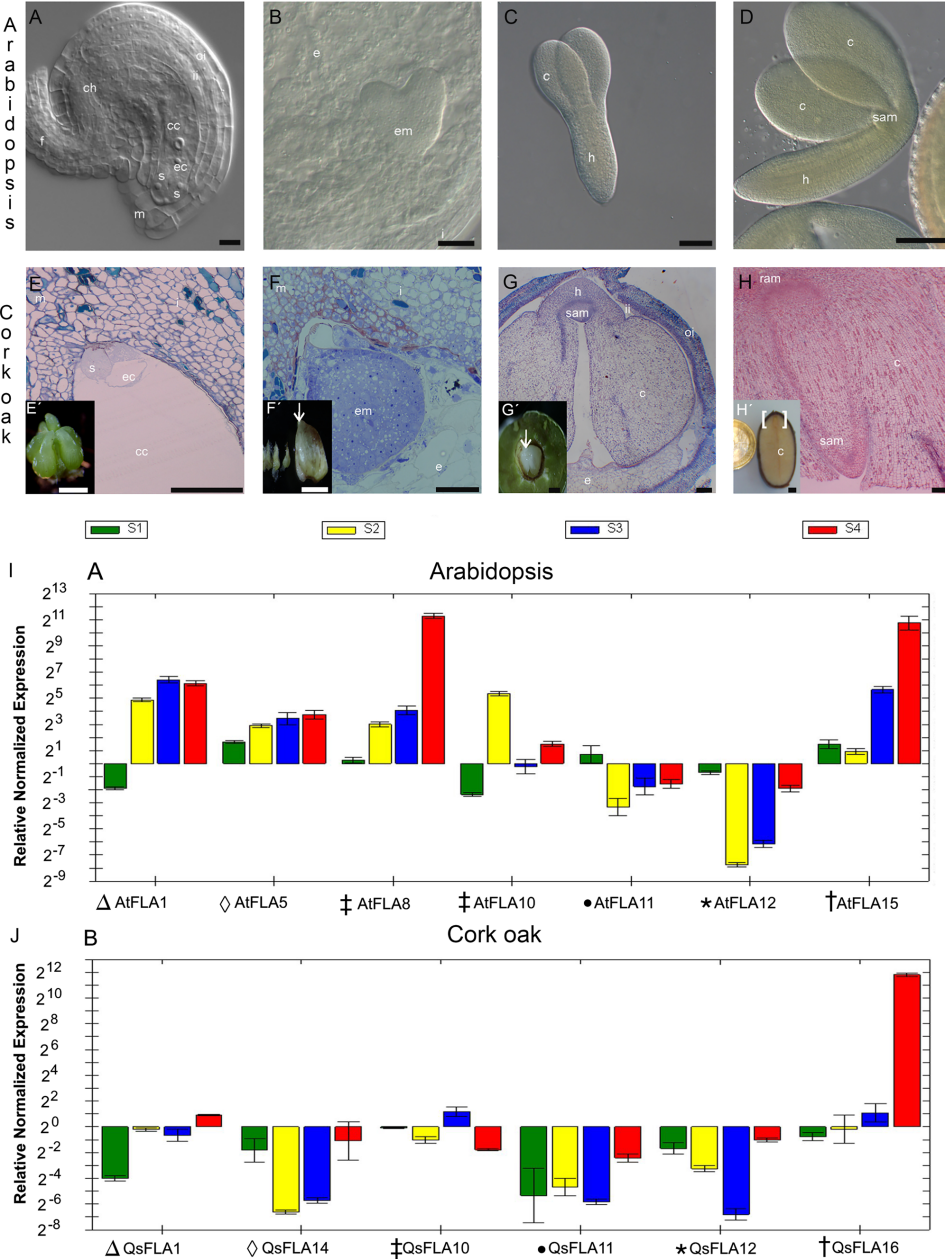
Expression of selected orthologous FLA genes of Arabidopsis and cork oak during seed/embryo development. Gene expression was evaluated in specific stages of embryo development. Stage 1 (S1): unfertilized mature ovules of Arabidopsis (**A**) and cork oak (**E**). Stage 2 (S2): whole seeds containing embryos from globular until heart-shaped stages (**B**, **F**). Stage 3(S3): torpedo to linear cotyledon stage embryos (**C**, **G**). Stage 4 (S4): walking stick to mature embryos (**D**, **H**). The qPCR expression analysis of *AtFLA1*, *AtFLA5*, *AtFLA8, AtFLA10*, *AtFLA11*, *AtFLA12* and *AtFLA15* (**I**) and their putative orthologs *QsFLA1*, *QsFLA14, QsFLA10*, *QsFLA11*, *QsFLA12* and *QsFLA16* (**J**), respectively, was performed with three biological replicates each. Bars represent standard deviation between replicates. c, cotyledons; cc, central cell; ch, chalaza; e, endosperm; ec, egg cell; em, embryo; f, funiculus; h, hypocotyl, i, integument; ii, inner integuments; m, micropyle; oi, outer integuments; RAM, root apical meristem; s, synergid cells; SAM, shoot apical meristem; v, vasculature. Arrow in **F′**: fertilized embryo sac. Arrow in **G′**: cork oak embryo. Parentheses in H: 5 mm section of cork oak embryo samples. Scale bars: 100 µm (**A**, **B**, **E**–**F**); 5 mm (**C**, **D**, **E′**, **F′**, **G′**); 1 cm (**H′**)

A gene expression assay was performed to find similarities in gene regulation between the putative orthologs found by phylogenetic analysis, at equivalent developmental stages in both Arabidopsis and cork oak, using the mature leaf as a reference sample. Stage 1 (S1) consists of unfertilized ovules in Arabidopsis (Fig. 6A) and in cork oak (Fig. 6E). In cork oak, only one ovule reaches maturity, despite the formation of up to six morphologically equivalent ovules (Fig. 6E, inset). Stage 2 (S2) includes ovules with the respective integuments (i), containing the younger stages of embryo development from globular to heart-shaped embryo. In cork oak, the swelling of the ovule (Fig. 6F′ arrow) is indicative that the fertilization has occurred. This morphological feature aided in the isolation of fertilized ovule from aborted ones. In Arabidopsis (Fig. 6B), the ovule fertilization event is more cryptic, and the growth accompanying the embryo development is slower. Stage 3 (S3) consists of linear embryos only, extracted from the developing seeds (Fig. 6C, G). In both species, despite the overall size discrepancies, the shoot meristematic regions (SAM), cotyledons (*c*) and vasculature primordial (*v*) are well developed. In cork oak, the suspensor is small and the embryo remains shallow, growing slowly over the endosperm (Fig. 6F). Stage 4 (S4) contains isolated mature embryos without the seed coat. Arabidopsis mature embryos (Fig. 6D) are bent with long, well-vascularized, hypocotyl. The SAM is well developed but rudimentary, whereas in cork oak the embryos are straight, with minimal hypocotyls and enormous cotyledons.

In the selected developmental stages, the Arabidopsis expression pattern for the chosen FLAs was in accordance with the microarray-based heat maps (Fig. 2B–D), and the expression pattern in the cork oak putative orthologs was in most cases similar. AtFLA1 is expressed at an increasing rate across the four stages of seed development (Fig. 2B). The expression of its cork oak ortholog, QsFLA1 (LOC112015936), also shows a somewhat similar to that of AtFLA1 increase across the seed development (Fig. 6I, J).

Our study showed that AtFLA5 was predominantly expressed in the ovule, increasing its expression up to the linear embryo stage (S3) and reaching its expression maximum at the mature embryo stage (S4). The expression pattern of its putative ortholog in cork oak, QsFLA14 (LOC112017397) (Fig. 2A), showed, however, a marked difference since it was found to drop its expression between S1 and S2 before increasing its expression toward the mature stage S4.

AtFLA8 and AtFLA10 showed very distinct expression patterns. The expression pattern in the cork oak closest relative, QsFLA10 (LOC112008859), was more similar to AtFLA10 expression pattern, than to AtFLA8, except for stages S2, S3 and S4. In these stages, the gene expression levels were inversed between the two species. The expression pattern of AtFLA11/QsFLA11 (LOC112005478), AtFLA12/QsFLA12 (LOC112031083) and AtFLA15/QsFLA16 (LOC112003793) was highly similar across all stages.

## Discussion

### AtFLAs and its orthologs

Angiosperms are the most diverse and widespread group of land plants. They diverged from the gymnosperms during the Triassic period, and during the Cretaceous period, a great burst in biodiversity led to the emergence of over 300,000 species distributed across 416 families. Charles Darwin referred to the angiosperm diversity explosion as “an abominable mystery.” Despite the evolutionary divergences accumulated in the past 200 million years, the A, B, C and D FLA groups remained conserved in angiosperms and homologs can be easily identified to the majority of FLAs in these four species. A hint of a FLA group-associated role can be devised from there diversity and number in different plant growth habits and developmental processes.

Group A FLAs have been suggested to play a role in secondary cell wall assembly and may physically interact with the cellulose synthase complex, as suggested by previous work on poplar hemp and cotton (Huang et al. 2013; Bygdell et al. 2017; Guerriero et al. 2017). The observed abundance of group A FLAs in poplar and cork oak, especially AtFLA12 and AtFLA11 orthologs, may reflect this functional specificity toward the formation of woody structures.

Group B FLAs are by far the most homogenous group forming a neat tight cluster. In Arabidopsis, according to the available microarray data, their expression patterns seem to be coordinated in the embryo development; the present analysis confirms the maintenance of the expression pattern between two group B orthologs, AtFLA15 and QsFLA16. Whether this is true to other genes in group B remains to be determined.

FLAs of group C seem to be more related to developmental roles in reproduction. Group C forms two distinct subclades separating the AtFLA5/AtFLA3 and the AtFLA8/AtFLA10 paralog pairs. AtFLA5 and AtFLA3 are predominantly expressed during the pollen development and have been related to its maturation (Li et al. 2010). The other group C subclade reaches important levels during seed development in Arabidopsis.

FLAs of group D are by far the most diverse, forming two main subclusters. Two of the clusters that stand out, by homologs number and divergence, are the AtFLA4- and the AtFLA21-containing clusters. Both of them seem to be involved in specific processes. AtFLA4 has been related to salt stress tolerance and root development. AtFLA21 and its homologs are specifically expressed during pollen maturation (Li et al. 2010; Seifert 2018). Putative orthologs of AtFLA4 can be found in all four species, whereas for AtFLA21, putative orthologs are not so obvious, perhaps reflecting pollen developmental specificities in these species.

### AtFLAs may have important roles during seed development

The presence of AGP-specific epitopes has been previously associated with diverse developmental processes, including plant reproduction and embryo development (Coimbra et al. 2007; El-Tantawy et al. 2013). The available microarray data of Arabidopsis FLA genes showed that the vast majority of them are expressed during seed development, some of which seem to be expressed in a stage and/or organ-specific manner, suggesting specific roles in this process.

Group B FLA genes (AtFLA15, 16 and 18), according to the microarray data, seem to be regulated in a similar manner. Throughout Arabidopsis embryo development, all Group B FLAs reach their maximum expression during the mature green stage. Apart from this particular stage of development, FLA expression in Arabidopsis seems to be unrelated to the proposed group classification. For instance, group C AtFLA1 is expressed at greater levels in the green mature stage and in the embryo, rather than in the seed coat. The AtFLA1 closest relative, AtFLA2, has a different expression pattern, being absent from the seed development. AtFLA10 is closely related to AtFLA8, but seems to be specifically expressed in the seed coat during the heart-shaped embryo stage, whereas AtFLA8 expression decreases from the preglobular stage onward. Although phylogenetically close, AtFLA8 and AtFLA10 are differentially regulated. Sequence homology seems to have little to do with the expression pattern, since closely related FLAs have distinct expression patterns. AtFLA11 and AtFLA12, two similar gene members in group A known to interact with cell wall-modifying proteins, are both expressed during embryo development in a similar fashion, but have a distinct expression pattern during the seed coat development. AtFLA11 and AtFLA12 are differentially expressed in these two structures, possibly contributing to its development. The results support the idea that Arabidopsis FLAs are tightly regulated during seed development and may play important roles in this process.

### Arabidopsis FLAs and its cork oak orthologs possible functions

Despite great differences in ovary structure, the ovules of Arabidopsis and cork oak are of the polygonum type, having embryo sacs with two synergid cells supporting one egg cell, a large diploid central cell and three antipodal cells, all surrounded by two integuments. Two relatively thin inner and outer integuments protect the Arabidopsis embryo sac, whereas in cork oak thick multilayer integuments surround the embryo sac.

Arabidopsis gynoecium harbors up to 60 ovules, the majority of which complete the developmental process up to maturity. In cork oak, only one ovule reaches maturity and is receptive, despite the formation of up to six morphologically equivalent ovules.

The Arabidopsis embryo forms a long suspensor that pushes the embryo proper deeply into the endosperm. In cork oak, the suspensor is less developed and the embryo remains shallow growing slowly over the endosperm. The embryo axis is short with an underdeveloped hypocotyl when compared to the Arabidopsis linear embryo. Arabidopsis mature embryos are bent with a long hypocotyl with a central procambium, the SAM is well developed but rudimentary, while in cork oak the embryos are linear, with minimal hypocotyl and very large cotyledons.

The expression results of the FLA genes tested by qPCR were in accordance with the microarray-based heat maps, and the expression pattern of the cork oak putative orthologs of Arabidopsis genes was in most cases similar. AtFLA1 was expressed at increasing levels during the four stages of seed development, which is in agreement with the respective microarray data, and the expression of QsFLA1 (LOC112015936) was to some extent similar, showing a tendency for increased transcript levels with embryo maturation.

AtFLA5 has no available data in microarray assays, and our study shows that AtFLA5 is expressed in the ovule, reducing its expression up to the linear embryo stage but recovering its expression at the mature embryo stage. The expression pattern of its closest putative ortholog in cork oak QsFLA14 (LOC112017397) was poorly expressed in all stages of embryo development.

AtFLA8 and AtFLA10, two group C FLAs with high amino acid sequence identity show very distinct expression patterns. Maybe the regulatory regions were highly modified during duplication of AtFLA8 and AtFLA10, or those two genes may be the result of evolutionary convergence. The expression pattern of cork oak putative ortholog (QsFLA10, LOC112008859) bears no similarity with AtFLA8, but is somewhat similar to AtFLA10, except for stages S2 and S3. In these stages, the gene expression differences between the two species may be due to intrinsic developmental characteristics of the seed development. At stage S3, cork oak has thick cell walls and highly vascularized cotyledons, already accounting for the majority of the embryo mass. This characteristic may account for the increase in QsFLA10 expression during this stage. The expression pattern of AtFLA11/QsFLA11 (LOC112005478), AtFLA12/QsFLA12 (LOC112031083) and AtFLA15/QsFLA16 (LOC112003793) is highly similar across all stages. Most probably, the regulation for QsFLA11, 12 and 16 was preserved.

### Validating the transcriptome-based data for FLA8 and FLA10

Through phylogenetic inference, AtFLA10 and AtFLA8 appear to be paralogs. According to the microarray data presented, AtFLA8 and AtFLA10 have identical expression patterns during seed development which could be attributed to similar expression pattern in the embryo, rather than in the seed coat, where their expression is most dissimilar.

The analysis of the promoter activities revealed that in the unfertilized ovule, both genes have similar expression patterns (S1). However, AtFLA8 is mostly expressed in the chalaza and funiculus, while AtFLA10 is just in the chalaza. This difference helps to explain the distinct expression levels detected by qPCR, a great downregulation of AFLA10 comparing to AtFLA8.

From the globular to the heart-shaped stage (S2), qPCR results showed that although AtFLA8 and AtFLA10 were upregulated, compared to the leaf sample, AtFLA10 expression level was much higher than AtFLA8, somewhat in accordance with the microarray data. Between these stages, reporter protein signal was detected in the chalaza for both gene promoter sequences; however, AtFLA8 promoter drove GFP expression in the funiculus, while for AtFLA10 promoter, GFP signal was observed in the peripheral endosperm of the seed. Therefore, the higher AtFLA10 expression detected by qPCR may be explained by the expression of AtFLA10 in the peripheral endosperm as it starts to divide. The microarray data indicated that AtFLA10 transcripts were highly expressed in the seed coat at the heart-shaped stage maybe due to peripheral endosperm contamination. Interestingly, the microarray data pointed that AtFLA8 expression maximum occurs in the globular embryo. However, in our experiments, it was not possible to observe GFP expression in the pAtFLA8:GFP globular embryos.

In S3 and S4 samples, only the embryos from heart-shaped to torpedo and from walking stick to mature stages were used for RNA extraction, respectively. In S3, a slight upregulation of AtFLA8 was observed, whereas for AtFLA10 a great downregulation was detected. The results obtained with the reporter gene experiments reflect this drastic difference in gene expression: It was possible to detect GFP signal in the hypocotyl cells of pAtFLA8:GFP heart-shaped to linear cotyledon embryos, while pAtFLA10:GFP embryos never presented any fluorescence. Regarding S4, the major upregulation observed for AtFLA8 does not seem to be in accordance with the GFP expression pattern. Considering that GFP is detected in the hypocotyl cells surrounding the developing vascular bundle, as the embryo grows the number of cells that express the construct decreases, compared to the total number of cells of the embryo. Therefore, the AtFLA8 transcript may become diluted in the sample.

Comparing the seed coat microarray data with the results obtained from the AtFLA8 and AtFLA10 promoter analyses, it is possible to note some dissimilarity. AtFLA8 transcript level is higher at the preglobular stage and greatly decreases in the mature green seed. Between the preglobular and globular seed stages, pAtFLA8:GFP seeds showed fluorescent signal just in the chalaza, while in later stages GFP was found across the seed coat. Therefore, the number of cells that express the gene should be higher in later stages of development. AtFLA10 transcript abundance reaches its maximum at the heart-shaped stage and is the lowest in the mature green stage. pAtFLA10:GFP seeds showed GFP expression in inner integuments of the seed coat at the heart-shaped stage and in the outer layer of the seed coat in more mature stages. In fact, the signal in the outer layer of the seed coat appeared more intense than the signal observed for the heart-shaped stage, which does not match the information extracted from the microarray data.

Importantly, based on seed microarray data AtFLA8 and AtFLA10 have identical expression patterns in seeds. It could be assumed that the embryo rather than the seed coat contributed the most to that outcome, but that does not seem to be the case. Indeed, the expression of pAtFLA8:GFP and pAtFLA10:GFP in both embryos and seed coat is quite different.

Our results highlight the relevance of performing expression assays and characterize the expression pattern of genes to validate transcriptome-based data.

## Concluding remarks

Our results show FLA group structure conservation across selected angiosperms and support the idea that Arabidopsis FLAs are tightly regulated during seed development and may play important roles in this process.

Moreover, the expression pattern of AtFLA5 does not correspond to its closest putative ortholog in cork oak QsFLA14. AtFLA8 and AtFLA10, two group C FLAs with high amino acid sequence identity show very distinct expression patterns.

Our results highlight the relevance of performing expression assays and characterize the expression pattern of genes to validate transcriptome-based data.

### Author contribution statement

MC performed the bioinformatics studies, SCP, MC performed the qPCR experiments, AMP made the GFP constructs SCP, JS executed the GFP expression tests, and LGP and SC programmed the work, wrote and reviewed the manuscript.

## Electronic supplementary material

Below is the link to the electronic supplementary material.

Supplementary material 1 (JPEG 782 kb)

Supplementary material 2 (DOCX 34 kb)
