# Supplementary figures and images for: In silico and expression analyses of fasciclin-like arabinogalactan proteins reveal functional conservation during embryo and seed development
Source: Plant Reprod. 2019 Sep 9;32(4):353–70. doi: 10.1007/s00497-019-00376-7 (PMC6820600; doi:10.1007/s00497-019-00376-7)

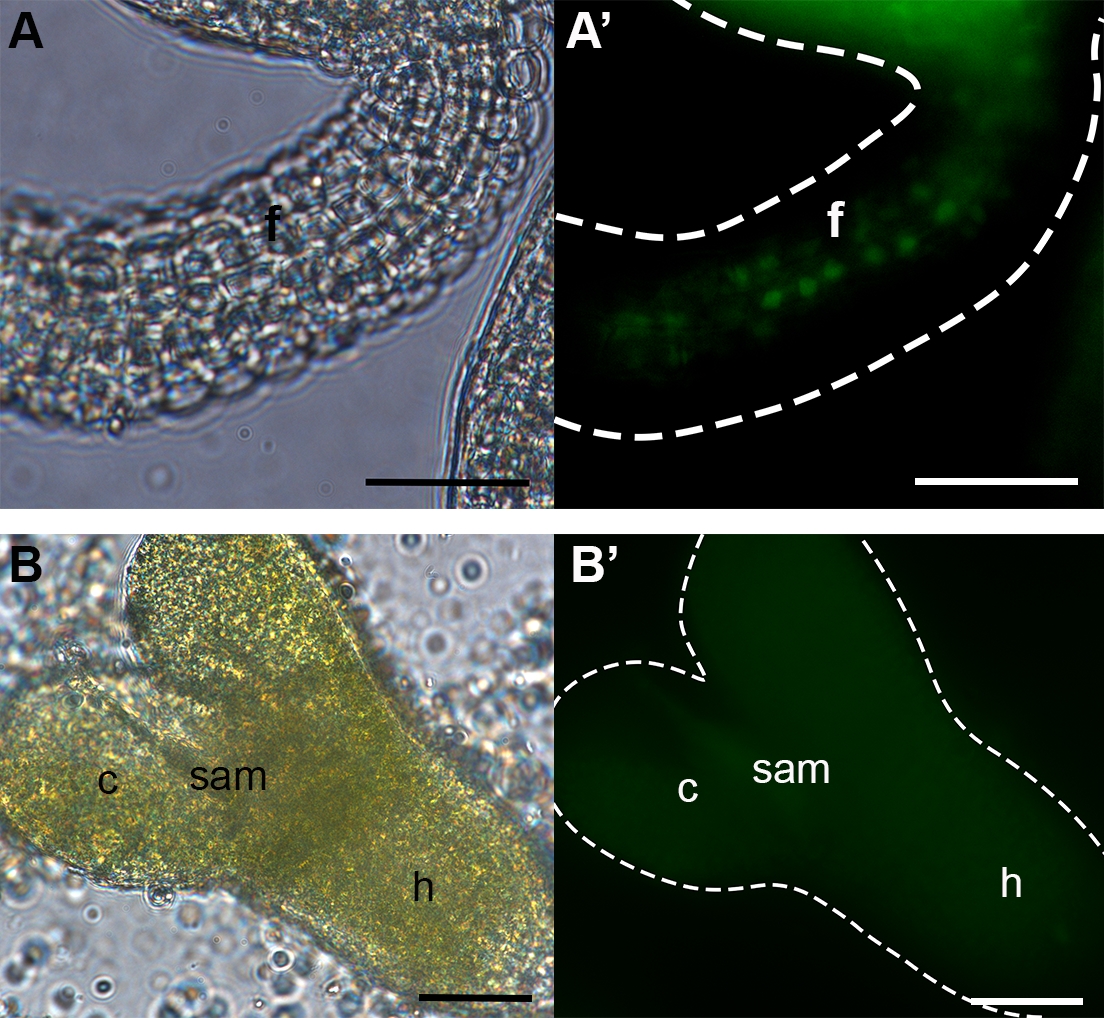

Supplement: Supplementary file 1 — Supplementary material 1 (JPEG 782 kb) [file 497_2019_376_MOESM1_ESM.jpg]
