# Supplementary material for: In silico and expression analyses of fasciclin-like arabinogalactan proteins reveal functional conservation during embryo and seed development
Source: Plant Reprod. 2019 Sep 9;32(4):353–70. doi: 10.1007/s00497-019-00376-7 (PMC6820600; doi:10.1007/s00497-019-00376-7)
Supplement: Supplementary file 2 — Supplementary material 2 (DOCX 34 kb) [file 497_2019_376_MOESM2_ESM.docx]

Fasciclin-like Arabinogalactan Proteins: structure and expression patterns are highly conserved across angiosperms

Mário Costa^1,2^, Ana Marta Pereira ^3^, Sara Cristina Pinto ^1,2^, Jessy Silva ^1,2^, Luís Gustavo Pereira^1,2^, Sílvia Coimbra ^1,2*^

^1^Departamento de Biologia, Faculdade de Ciências da Universidade do Porto, Porto, Portugal.

^2^GreenUPorto Sustainable Agrifood Production Research Centre, Porto, Portugal. ^3^Dipartimento di Bioscienze, Università Degli Studi di Milano, Milano, Italy.

*** Correspondence:**Sílvia Coimbra
[scoimbra@fc.up.pt](mailto:scoimbra@fc.up.pt)

Supplementary Material

**Supplementary table S1.** *Arabidopsis thaliana* 21k Affymetrix genechip array probe identification.

| **Name** | **Locus** | **Probe ID** |
| --- | --- | --- |
| FLA1 | AT5G55730 | 248074_at |
| FLA2 | AT4G12730 | 254785_at |
| FLA3 | AT2G24450 | 257392_at |
| FLA6 | AT2G20520 | 263376_at |
| FLA7 | AT2G04780 | 263628_at |
| FLA8 | AT2G45470 | 251395_at |
| FLA9 | AT1G03870 | 265066_at |
| FLA10 | AT3G60900 | 251394_at |
| FLA11 | AT5G03170 | 250933_at |
| FLA12 | AT5G60490 | 247638_at |
| FLA13 | AT5G44130 | 249037_at |
| FLA14 | AT3G12660 | 257691_at |
| FLA15 | AT3G52370 | 256673_at |
| FLA16 | AT2G35860 | 263942_at |
| FLA18 | AT3G11700 | 259072_at |
| FLA19 | AT1G15190 | 262606_at |
| FLA20 | AT5G40940 | 249323_at |
| FLA21 | AT5G06920 | 250652_at |

**Supplementary table S2.** Primer list for the Real Time RT-PCR experiment with the selected FLAs in *Arabidopsis thaliana*.

| Name | Locus | Primers |
| --- | --- | --- |
| FLA1 | AT5G55730 | F GGATGCAAAGTGTTTGCTGA  R ACAGCGAGGAAATCGAGAAA |
| FLA5 | AT4G31370 | F TTCTCTCCTCTCCCTCACCA  R GATGGCTGCAATGAGCTTGG |
| FLA8 | AT2G45470 | F CACTCCTCGCCTTCACTTTC  R ATTGCGCCATTGTTAAGGAC |
| FLA10 | AT3G60900 | F CGTCGTCAAAAATGCTCTCA  R CCTTTGAGATCGGTGACGTT |
| FLA11 | AT5G03170 | F GGCGATGATGGAGGAGATTC  R CAATGGCTGCAACGGTAGTG |
| FLA12 | AT5G60490 | F CAGGACCCACAAACGTTACC  R CGGAGTTGTTTAGTTGGCCG |
| FLA15 | AT3G52370 | F CGTGTCGGAAGGTTACGTTT  R TGAGGAAACCGGAGTGAATC |
| ACT2 | AT3G18780 | F TCGGTGGTTCCATTCTTGCT  R CGGCCTTGGAGATCCACAT |
| ACT8 | AT1G49240 | F CTCAGGTATTGCAGACCGTATGAG  R CTGGACCTGCTTCATCATACTCTG |

**Supplementary table S3.** Primer list for the Real Time RT-PCR experiment with the selected FLAs in *Quercus suber.*

| Name | Locus | Primers |
| --- | --- | --- |
| FLA1 | XM_024048405.1 | F TCAAAACGACAATGCAGCTC  R CAGACGGTTATGGTGGTGTG |
| FLA10 | XM_024041212.1 | F GACGGCTGATAAGGGTTTGA  R GTGTCGATCGGATCCTTTGT |
| FLA11 | XM_024037729.1 | F ACTTCTGCTCAGTCTCCACC  R TGGTGCAAAGATGGTAAGGC |
| FLA12 | XM_024063762.1 | F CTTCCAAACCGTGAGTAACCC  R CAAGACCAGAGGAGATGTTCAC |
| FLA14 | XM_024049872.1 | F GAAACCAAACCCATCACCAC  R CAGCAGAGGCTGATACACCA |
| FLA16 | XM_024036006.1 | F GGAGCGAAGATGGTGATGTT  R CATTGCCTCAAAACCCAGTT |
| ACT7 | XM_024037498.1 | F GCTGGTCGTGATCTAACTG  R CTTTGCAGTCTCCAACTCCT |
| PP2AA3 | XM_024062388 | F GGGTTCCCAACATCAAGTTC  R TGACCTGATCACTTGACTGC |

**Supplementary table S4**. Primer list for FLAs promoter amplification and use in GFP fusions.

| Name | Locus | Primers |
| --- | --- | --- |
| FLA8 | AT2G45470 | F CACCGACTGTTAACTCCTCTGTCCAG  R GATGAGACGGTTTTGAGTTTTTTCAAGTGTCA |
| FLA10 | AT3G60900 | F CACCCTTCGTCGTTGTTTTTGACCAG  R ATTTGGAAGGTTGAGTTGAACTG |

**Supplementary figure S1.** GFP expression driven by AtFLA8 promoter in the funiculus of in the unfertilized ovule **(A-A’)** and by AtFLA10 promoter in the late heart-stage embryo **(B-B’)**. c, cotyledon; f, funiculus; h, hypocotyl; sam, shoot apical meristem. Scale bars: 100 µm.
